# From Guidelines to Social Media: A Content Analysis of Trauma-Informed Care on YouTube

**DOI:** 10.3390/bs15030340

**Published:** 2025-03-10

**Authors:** Aysha Jawed, Mollie Young, Sayyed Matin Zarkesh Esfahani

**Affiliations:** 1 Department of Pediatrics, Johns Hopkins University School of Medicine, Baltimore, MD 21205, USA; myoung49@jhmi.edu; 2 Johns Hopkins Children’s Center, Baltimore, MD 21287, USA; 3 Department of English, University of Maryland, College Park, MD 20742, USA; szarkesh@terpmail.umd.edu

**Keywords:** trauma-informed care, resilience, safety, collaboration, healing, organizational approach

## Abstract

Trauma-informed care is an increasingly trending clinical and organizational approach globally. Multiple guidelines exist on implementing trauma-informed care across healthcare systems, behavioral health programs, academic institutions, and prisons, among other settings. Although many studies have assessed the implementation of trauma-informed care guidelines and the integration of training into curricula for healthcare providers, workforces, and in clinical practice with individuals and communities, there have been no studies previously conducted to date on assessing the existing state of coverage on trauma-informed care across social media to inform future, actionable interventions. This represents a critical gap in research and practice given the increasingly prevalent utilization and accessibility of information online, especially via a multitude of social media platforms. This study is the first to assess the sources, format, and content across one of these social media platforms on YouTube. Content on trauma-informed care was examined through conducting a descriptive, observational study to determine the depth and breadth of content that was widely covered and uncovered across the top 100 widely viewed videos. Findings revealed that most of the content was published by professional, nongovernmental sources. A wide range of resources and strategies was presented on social media for utilizing trauma-informed care across diverse settings on individual and community levels. The five principles of trauma-informed care (safety, trustworthiness, collaboration, empowerment, and choice) were heavily reviewed among the widely viewed videos. A multitude of benefits was presented in terms of implementing trauma-informed care on both micro and macro levels. Social determinants of health were not widely covered but formed some of the stressors and triggers examined among the videos. DEI principles were also scantly covered across the videos. Several clinical and organizational implications are presented. Recommendations to integrate widely covered and uncovered content as targets for intervention in informing future trauma-informed approaches are proposed.

## 1. Introduction

Trauma-informed care is a rising, trending approach that transcends healthcare, academic, criminal justice, and other organizational contexts, including workforces, worldwide. This care delivery model has demonstrated effectiveness in treatment planning, developing thriving organizations, strengthening workforces, creating a path for healing for patients. and cultivating academic success among students (Substance Abuse Mental Health Services Administration, 2023; Forkey et al., 2021; United Nations Children’s Emergency Fund, n.d.). Multiple components are at the heart of trauma-informed care. These include the core principles of safety, trustworthiness, collaboration, empowerment and choice (Berring et al., 2024). In addition, social determinants of health and diversity, equity and inclusion (DEI) components are critical factors in implementing trauma-informed care across various contexts (Morgan et al., 2023).

Multiple trauma-informed care guidelines have been developed in the United States as well as globally for diverse patient populations. The Substance Abuse and Mental Health Services Administration (SAMHSA) is a governmental organization in the United States that has developed a set of guidelines for the delivery of trauma-informed care in behavioral health (2023). The American Academy of Pediatrics is a professional society that has also published guidelines on providing trauma-informed care to children and adolescents as a pathway for healing, optimizing functioning, and creating the next generation of healthier communities through bolstering resilience among children (Forkey et al., 2021). The United Nations has also been at the forefront of publishing guidelines on care delivery utilizing principles of trauma-informed care (United Nations Children’s Emergency Fund, n.d.). In addition, many universities, for profit and non-profit organizations, as well as federal, state and local governments across many countries, have also created guidelines for trauma-informed approaches worldwide (Emsley et al., 2022; Maynard et al., 2019; Bitanihirwe & Imad, 2023; Trofimoff et al., 2024; Brown et al., 2021; Hargrove et al., 2024; Henshaw, 2022; Davidson, 2017). Notably, the ease of adaptability of trauma-informed care across contexts, along with the consistencies and commonalities across guidelines, yield significant potential for building a trauma-informed global community.

Although trauma-informed care has been extensively studied and reviewed worldwide through the development and implementation of guidelines and organizational and clinical approaches, as well as in strengthening workforces through the delivery of trainings and curriculum development for staff education, there have been no studies that have examined the current state of coverage for trauma-informed care on social media. Some of the training programs are promoted on social media via Facebook, YouTube, Instagram, X (formerly known as Twitter), and TikTok, among more digital spaces. It follows that time is of the essence to determine whether social media could be yet another mode of communication and knowledge dissemination across contexts in expanding the reach, awareness, and cues for action in integrating the principles of trauma-informed care across different systems.

Based on a recent study published by the Pew Research Center, YouTube, Facebook, Instagram, TikTok, and X are among the most widely utilized social media platforms (Pew Research Center, 2024). Given that the literature is nonexistent on trauma-informed care coverage across any of these digital spaces, this study sought to begin filling this gap in the existing body of literature. Since YouTube is publicly accessible, this is the digital space that forms the setting for the focus of this study. It follows that the goals of this study are the following: (1) to identity the sources and formats for widely viewed videos pertaining to trauma-informed care on YouTube; (2) to examine the depth and breadth of content across this sample of videos; (3) and to present clinical and organizational implications along with recommendations for future research and practice in harnessing the potential of trauma-informed care in informing sustainable interventions across multiple contexts.

## 2. Materials and Methods

This study was descriptive and cross-sectional in nature, involving content analyses of widely covered content pertaining to trauma-informed care on YouTube. Observational data were collected at one conceptual point in time from YouTube. During December 2024 to January 2025, the browser history on computers for all of the authors was cleared. The authors piloted search strategies with key words and phrases (e.g., trauma-informed care, trauma-informed principles, trauma-informed approach) to determine which ones yielded the most relevant videos as well as the highest view counts and cumulative views for the top 30, 60, and 100 videos pertaining to trauma-informed care. Ultimately, the key terms that met each of these criteria were “trauma-informed care”. The results were then filtered based on view count, and the authors stored the URLs for the top 100 widely viewed videos in a separate document. Duplicate URLs were deleted and replaced, thereby ensuring that solely one URL for each video formed the unit of coding and analysis.

A codebook was developed by the researchers based on a comprehensive review of the literature, guidelines and approaches from authoritative, credible, and expert sources globally that included the Substance Abuse and Mental Health Services Administration (SAMHSA), Centers for Disease Control and Prevention (CDC), National Institutes of Health (NIH), American Academy of Pediatrics (AAP), and the United Nations. Each researcher on the team viewed and coded all of the videos in this sample from December 2024 to January 2025. This study established intra- and inter-rater reliability across the coding for each researcher (AJ, MY, and SMZE) and among the research team, respectively. Specific information that was reviewed and coded for each video as the unit of analysis include the following: (a) source of the video upload, (b) the format of video, (c) the number of views for each video, (d) the length in minutes of each video, (e) the year of video upload, and (f) the content of each video (described further in this section). This study did not require IRB approval given that these videos were publicly available online.

### 2.1. Eligibility Criteria

Solely videos in English were reviewed in the study. It follows that any videos which were either narrated or presented any form of written or verbal content in a different language were ultimately excluded from coding and analysis. Content for all videos in this sample focused on trauma-informed care. The researchers viewed each video in its entirety, which represented the unit of analysis. There were no minimum or maximum length requirements for the videos in this sample.

### 2.2. Measurements and Coding Specifications

As aforementioned, the basic information included in the instrument were the following: coder, assigned video identification number, upload date of the video, coding date of the video, length of the video (in minutes), the number of views, and the title of the video. The next part of the instrument included three sections for the sources of upload, format, and content. Specifically, content encompassed multiple content categories consisting of many variables (reviewed below), which were all coded dichotomously (i.e., either yes or no) to depict presence or absence in each video.

The source of upload was mutually exclusive for each video and, in turn, coded into one of the following four categories: organizational, consumer, governmental, and other sources. The categories for video format included Documentary; Interview; Demonstration/Experiment; Talk by Professional; TV Talk Show/Discussion Panel; Animation; Still Images; News Report with Anchor; V-blog; Advertisement; Testimonial/Story; Multiple Formats; and Other Formats. Ultimately, the following 16 content categories were created for this codebook: (a) principles of trauma-informed care; (b) value-based considerations; (c) DEI considerations; (d) social determinants of health; (e) stressors and triggers; (f) stages of development; (g) settings; (h) audiences; (i) fields; (j) resources and strategies; (k) professional roles; (l) community and national groups; (m) barriers; (n) specialized considerations; (o) benefits; and (p) open-ended comments on misinformation or disinformation depicted in the video. Conceptualization of the codebook involved the development of these content categories to account for the comprehensive range of mediating and moderating factors, risk and protective factors, interventions, and biopsychosocial considerations surrounding trauma-informed care.

### 2.3. Demonstration of Intra- and Inter-Rater Reliability

In this study, the researchers demonstrated both intra- and inter-rater reliability of the observational data from the content coded across all of the videos in this sample of widely viewed videos. To demonstrate intra-rater reliability, the researchers (AJ, MY, and SMZE) utilized a random number generator which entailed randomly selecting ten videos and then recoding them within two weeks of the original coding. This analysis accounted for all of the dichotomously coded (Yes versus No) content variables in the instrument. Intra-rater reliability was found to be high (Kappa = 0.94). Inter-rater reliability was also demonstrated from coding completed by the researchers (AJ, MY, and SMZE). A random number generator was also utilized to select ten of the coded videos for analysis across the constellation of variables. The researchers subsequently reviewed coding responses to resolve any discrepancies in this coding instrument. Inter-rater reliability was found to be high (Kappa = 0.93).

### 2.4. Statistical Analysis

Composite statistical analyses were conducted in this study (Song et al., 2013). These included computing descriptive statistics for all of the variables in the instrument. Observational data for the widely viewed videos were analyzed through computing frequencies and percentages of the source, format, number of views, length, and content of each video. For each variable within each content category, the number of videos accounting for the specific content (variable) was first uncovered. The total number of views from these videos covering this respective content was subsequently determined. The proportion of total cumulative views was next found by dividing the total number of content-related views from this subset of videos by the cumulative views for the entire sample of the widely viewed videos in this study (n = 16,183,837 views). These analyses were conducted for variables across each of the content categories for the entire coding instrument. All analyses were conducted using Microsoft Excel and Statistical Package for the Social Sciences (SPSS, version 29).

## 3. Results

The total number of views for the sample of the 100 most widely viewed videos was 16,183,837. The view counts ranged from 1023 to 7,690,983. These widely viewed videos were posted between 2011 and 2024. The lengths of the videos spanned 0.52 min to 60.7 min. The median length of the widely viewed videos was 2.64 min. The interquartile range for the sample ranged from 1.77 min to 3.95 min.

### 3.1. Stylistic and Qualitative Features of Videos

Many of the videos featured practices shared by deliverers of care or stakeholders (e.g., healthcare providers, educators, administrators) in group settings or individually. Three videos in the top ten widely viewed videos encapsulated content that drew in viewers and accounted for a multitude of formats and case examples. In one of these videos, a trauma-informed psychologist provided case examples with patients who have endured adverse childhood experiences (ACEs), grief/loss, and violence. In each case, different principles of trauma-informed care (safety, trust, collaboration, choice, empowerment) were reviewed to provide coaching, counseling, and problem-solving to support each patient. The translatable nature of trauma-informed approaches for each of these patients who were all at different stages in their lives was also reviewed, lending strength to the adaptability and poignancy of trauma-informed care across contexts.

Children as a patient population affected by trauma (specifically adverse childhood experiences) were also widely covered in this sample of 100 videos. Among the top ten widely viewed videos, one of the videos featured a case study of children affected by trauma, and the content reviewed involved multiple formats (e.g., still images, talks by professionals) and highlighted the significance of the department of social services, taking a trauma-informed approach to supporting children transitioning into foster or kinship care from the sequelae of trauma. The video itself was also integrated as an educational tool for caseworkers in local departments or social services. This video presented perspectives of a couple of deliverers of care (caseworkers) regarding the wraparound services they provided to children transitioning into foster or kinship care which integrated principles of trauma-informed care to support children working through their trauma and to improve functioning and engagement in school during this time. This video was community-focused and could likely be useful for other jurisdictions to utilize in training for caseworkers across departments of social services.

Trauma-informed schools were also widely covered across the sample of videos. Additionally, in the top 10 widely viewed videos in this sample of videos, a teacher presented strategies on how they arranged their classroom and found protected time to account for a range of expressive activities (art, group discussions, one-to-one discussions between teachers and students). In this video, the principal of the school also discussed how the school is seeking to take a trauma-informed approach across the school and featured the principal walking through the halls of the school and greeting students along the way who had endured trauma. The teacher in the video then discussed how these students were in their classrooms. This video also elucidated that the professional audiences heavily formed the bearers of content on trauma-informed care supported by verbal examples along with filming in the school and classrooms in a documentary style. The professional deliverers of content across the videos also strengthened the credibility of each video.

### 3.2. Sources

The majority of the widely viewed videos on trauma-informed care were posted by nongovernmental/organizational sources (*N* = 75), culminating in greater than 12 million views and nearly 76% of the cumulative views. Thirteen videos were published by other sources, generating greater than 3 million views and about 23% of the cumulative views. Seven videos were posted by the government, populating fewer than 200,000 views and about 1% of the cumulative views. Notably, only five videos were published by consumer sources, consisting of less than 100,000 views and less than 1% of the cumulative views.

### 3.3. Formats

There was a wide dispersion of formats presented across the widely viewed videos on trauma-informed care. Other formats were depicted across 49 videos, garnering more than 14 million views and about 88% of the cumulative views. Animation was presented in 30 videos, yielding almost 6 million views and nearly 36% of the cumulative views. Notably, although talks by professionals were covered in the majority of the videos (N = 70), their cumulative view count was substantially fewer (about 3 million views), representing about 18% of the cumulative views. Interviews were featured in three videos, culminating in greater than 1 million views (~9% of the cumulative views). Demonstrations/experiments were depicted in seven videos, generating approximately 1 million views (about 9% of the cumulative views). Still images were included in 20 videos, accounting for greater than 800,000 views (nearly 5% of the cumulative views). Testimonials were delineated in eight videos, garnering almost 700,000 views (~4% of the cumulative views). Multiple formats were portrayed across six videos, consisting of more than 500,000 views (about 3% of the cumulative views). TV talk shows and discussion panels were featured in one video, populating approximately 300,000 views and about 2% of the cumulative views.

### 3.4. Principles of Trauma-Informed Care

All of the principles of trauma-informed care were represented across the widely viewed videos. Safety was reviewed across 51 videos, garnering more than 5 million views (about 32% of the cumulative views). Trustworthiness was featured in 29 videos, yielding approximately 5 million views (about 30% of the cumulative views). Empowerment was depicted in 17 videos, culminating in more than 700,000 views (about 5% of the cumulative views). Collaboration was examined in 11 videos, constituting more than 600,000 views (about 4% of the cumulative views). Choice was included in 10 videos, generating ~400,000 views (about 3% of the cumulative views).

### 3.5. Value-Based Considerations

Value-based considerations were scantly covered across the widely viewed videos. Acceptability was reviewed in two videos, accounting for less than 100,000 views (<1% of the cumulative views). Sustainability was featured in one video, also yielding fewer than 100,000 views and <1% of the cumulative views.

### 3.6. Diversity, Equity and Inclusion

From a DEI perspective, solely inclusion was examined across the widely viewed videos, representing a miniscule number of views (N < 100,000) and also <1% of the cumulative views.

### 3.7. Social Determinants of Health

Social determinants of health were widely covered among this sample of videos. Exposure to violence was presented in 18 videos, garnering about 2 million views (~13% of the cumulative views). Race was covered in 11 videos, generating approximately 600,000 views (~4% of the cumulative views). Ethnicity was reviewed in eight videos, yielding also nearly 600,000 views (about 4% of the cumulative views). Socioeconomic status was depicted in three videos, culminating in almost 400,000 views (about 2% of the cumulative views). Similarly although the number of videos delineating housing stability were greater (N = 9), these videos generated a comparative number of views (~400,000), representing also nearly 2% of the cumulative views. Food insecurity was included in six videos, cultivating more than 200,000 views (<2% of the cumulative views). Access to transportation was featured in one video, constituting approximately 200,000 views (~1% of the cumulative views).

### 3.8. Stressors and Triggers

There were multiple stressors/triggers accounted for in the widely viewed videos. Trauma was covered in 78 videos, yielding more than 15 million views (about 94% of the cumulative views). Grief/loss was reviewed in eight videos, culminating in almost 8 million views (about 50% of the cumulative views). Psychiatric co-morbidities were presented in 31 videos, garnering nearly 6 million views (about 37% of the cumulative views). Adverse childhood experiences (ACEs) were examined in 33 videos, generating also approximately 6 million views (about 35% of the cumulative views). Violence was featured in 22 videos, cultivating more than 5 million views (about 34% of the cumulative views). Environmental adversity was depicted in 18 videos, accounting for more than 5 million views and nearly 33% of the cumulative views. Abuse was portrayed in 25 videos, yielding also more than 5 million views (about 32% of the cumulative views). Domestic violence was examined in 15 videos, populating approximately 5 million views (~31% of the cumulative views). Neglect was included in 17 videos, garnering more than 4 million views (about 27% of the cumulative views). Substance use disorders were covered in 20 videos, populating nearly 3 million views (about 18% of the cumulative views). Increased suicide risk was reviewed in eight videos, consisting of more than 2 million views (about 13% of the cumulative views). Sexual abuse was represented in 14 videos, culminating in approximately 2 million views (~13% of the cumulative views). PTSD was depicted in six videos, garnering almost 2 million views (about 11% of the cumulative views). Poverty was featured in 10 videos, yielding > 1 million views (about 8% of the cumulative views).

### 3.9. Stages of Development

The stages of development were also covered across the widely viewed videos. Children were reviewed in 34 videos, accounting for approximately 14 million views (about 90% of the cumulative views). Adults were featured in four videos, culminating in more than 8 million views (about 50% of the cumulative views). Adolescents were included in five videos, garnering almost 8 million views (about 50% of the cumulative views).

### 3.10. Settings

There was also a wide representation of settings across the widely viewed videos. Healthcare systems were presented in 24 videos, culminating in greater than 8 million views (>50% of the cumulative views). K-12 schools were included in 27 videos, garnering more than 4 million views (about 28% of the cumulative views). Academic institutions were covered in nine videos, yielding more than 400,000 views (about 3% of the cumulative views). Life skill programs were portrayed in five videos, generating almost 300,000 views (about 2% of the cumulative views). Prisons were depicted in six videos, accounting for nearly 200,000 views (about 1% of the cumulative views). Mental health clinics were represented in six videos, cultivating more than 100,000 views (<1% of the cumulative views).

### 3.11. Audiences

Multiple audiences were represented across the widely viewed videos. Patients were featured in 25 videos, culminating in nearly 9 million views (>50% of the cumulative views). Other clinical and ancillary staff were included in 23 videos, garnering also approximately 9 million views (>50% of the cumulative views). Doctors were portrayed in three videos, yielding almost 8 million views (about 50% of the cumulative views). Caregivers were depicted in five videos, cultivating about 8 million views (~50% of the cumulative views). Nurses were represented in six videos, generating nearly 8 million views (nearly 50% of the cumulative views). Police were delineated in 10 videos, accounting for almost 2 million views (~11% of the cumulative views). Teachers/educators were a part of 24 videos, constituting almost 2 million views (about 11% of the cumulative views). Students were featured in 13 videos, populating less than a million views (~6% of the cumulative views).

### 3.12. Fields of Practice

Several fields were included across the widely viewed videos. Healthcare was represented in 10 of the videos, culminating in greater than 8 million views and approximately 50% of the cumulative views. The legal field was included in 12 videos, garnering a substantially lower number of views (~300,000) and about 2% of the cumulative views. Social services were depicted in eight videos, populating less than 100,000 views (<1% of the cumulative views).

### 3.13. Resources and Strategies

There was also a wide dispersion of resources and strategies covered among the widely viewed videos. Self-regulation was reviewed in 19 videos, garnering more than 4 million views (about 28% of the cumulative views). Respond (Don’t React) was examined in 18 videos, generating nearly 4 million views (about 23% of the cumulative views). Training was included in 46 videos, accounting for more than 2 million views (approximately 16% of the cumulative views). Stress management was presented in 20 videos, populating more than 1 million views (about 8% of the cumulative views). Cultivating an emotionally and psychologically safe space was delineated in 23 videos, yielding less than a million views (~6% of the cumulative views). Trauma-informed workforce was covered in 13 videos, constituting approximately 900,000 views (~5% of the cumulative views). Coaching and mentoring were depicted in 14 videos, accounting for approximately 800,000 views (about 5% of the cumulative views). Community resources were reviewed in 10 videos, garnering almost 700,000 views (nearly 4% of the cumulative views). Screening was examined in 15 videos, cultivating almost 600,000 views (about 4% of the cumulative views). Active listening was included in seven videos, generating more than 500,000 views (about 3% of the cumulative views).

### 3.14. Professional Roles

Professional roles were scantly represented across the widely viewed videos. Leaders were covered the most across 13 videos, yielding about 400,000 views (~2% of the cumulative views). Although administrators were included in fewer videos (N = 7), these videos garnered a comparative number of views (~400,000) and about 2% of the cumulative views.

### 3.15. Community and National Groups

Multiple community and national groups were accounted for in the widely viewed videos. Healthcare systems were a part of 24 videos, generating more than 8 million views (>50% of the cumulative views). Non-profit organizations were reviewed in 15 videos, garnering more than 1 million views (about 10% of the cumulative views). Health departments were portrayed in two videos, yielding more than 300,000 views (about 2% of the cumulative views). Federal/national governments were included in six videos, populating more than 100,000 views (<1% of the cumulative views). A comparative five videos reviewed local governments, similarly generating >100,000 views and <1% of the cumulative views. Police were also comparatively featured in four videos, cultivating more than 100,000 views and also <1% of the cumulative views.

### 3.16. Barriers

Barriers were rarely covered across the widely viewed videos. Implicit bias and explicit bias were each featured in three videos, culminating in less than 200,000 views in total (~1% of the cumulative views).

### 3.17. Specialized Considerations

Specialized considerations were also scantly covered among the widely viewed videos. Developmental factors were reviewed in solely one video, garnering more than 400,000 views (about 3% of the cumulative views). Cultural factors were examined in seven videos, yielding nearly 200,000 views (about 1% of the cumulative views). Religious factors were included in two videos, accounting for <200,000 views (~1% of the cumulative views).

### 3.18. Benefits

A multitude of benefits were reviewed among the widely viewed videos. Mutual respect was covered in 13 videos, populating more than 8 million views (approximately 50% of the cumulative views). Although an increasing number of videos reviewed healing (N = 39), there were substantially fewer views generated (about 6 million), accounting for about 37% of the cumulative views. Increased safety was examined in 51 videos, culminating in more than 5 million views (about 34% of the cumulative views). Healthier relationships were portrayed in 34 videos, constituting more than 5 million views (about 32% of the cumulative views). Enhanced trust was delineated in 30 videos, cultivating approximately 5 million views and ~31% of the cumulative views. Effective treatment planning was reviewed in 13 videos, garnering almost 4 million views (about 24% of the cumulative views). Resilience was presented in 20 videos, culminating in approximately 4 million views (about 24% of the cumulative views). Thriving organization was included in 20 videos but yielded a substantially lower number of views (>800,000) and represented nearly 5% of the cumulative views. Increased empowerment/self-efficacy was examined in 17 videos, accounting for more than 700,000 views (about 5% of the cumulative views). Optimizing learning outcomes was presented in 16 videos, populating nearly 700,000 views (about 4% of the cumulative views). Productivity was portrayed in 13 videos, generating more than 600,000 views (4% of the cumulative views). Table 1, Table 2, Table 3, Table 4 and Table 5 provide a comprehensive breakdown of sources, format and pertinent content covered across the widely viewed videos on trauma-informed care.

## 4. Discussion

Most of the videos were posted by professional sources. Many of these videos accounted for a wide range of resources and strategies globally to integrate the principles of trauma-informed care across a multitude of settings that included healthcare systems, academic institutions, workplaces and much more. DEI and social determinants of health were not extensively covered in these widely viewed videos. Many of the videos included diverse formats in disseminating content on trauma-informed care. Multiple professional audiences alongside patients and students were represented across this sample of videos. The vast majority of the videos accounted for integrating principles of trauma-informed care in practices with children. Adverse experiences were the most widely covered stressors/triggers in heightening traumatic responses among diverse patient populations. Many benefits on both individual and community levels were presented from the implementation of trauma-informed practices across contexts.

As previously mentioned, most of the widely viewed videos on trauma-informed care were published by professional sources. Trauma-informed care is a paradigm shift that transcends a range of contexts. Its core principles have been integrated into the trainings of many deliverers of care in different workplace settings as well as into the nuts and bolts of care delivery with patients, students, community residents among more populations across the lifespan (Purtle, 2020; Burns et al., 2023; Gundacker et al., 2021; Steen et al., 2022). Among the widely viewed videos, several videos referenced different training workshops. In fact, continuing education programs to renew licensures for healthcare providers, teachers, and other clinical and educational staff were highly visible in this sample of videos, suggesting that trauma-informed care is a crucial part of sustaining professional practice with our communities. In addition, a multitude of resources and strategies were presented on both individual and community levels, many of which were also in professional settings. This further heightens the feasibility of implementing trauma-informed care in one or more ways that is adaptable and in line with the resources and needs of different care delivery models and institutions. It follows that drawing on one or more training workshops presented which integrate one or more of the resources and strategies reviewed across the widely viewed videos could help inform the acquisition of skills and concepts in building and sustaining trauma-informed workforces, programs, and communities globally.

Notably, although there are multiple guidelines published on trauma-informed principles across different settings, primarily in the healthcare context, these guidelines were not highly represented across the widely viewed videos. Among the ones that were posted by different for-profit and non-profit organizations, governmental organizations and professional societies, the content in these videos were in line with the principles of trauma-informed care, thereby representing an absence of misinformation and disinformation of content in this sample of videos and, in return, increasing the reliability of the depicted content. Many of these guidelines are published by governmental organizations. However, most of the videos in this sample were posted by primarily nongovernmental/organizational sources. These videos integrated diverse formats, resources, and stories from care providers. It follows that examining content posted by the government could be a starting point to determine whether there is opportunity to re-evaluate dissemination of content as the basis to heighten engagement via view counts, likes, tweets, shares and/or comments as engagement metrics. In addition, it is possible that forming collaborative spaces between federal, state and local governments with nongovernmental organizations on both community and global levels could provide opportunities to create robust content that is relatable, engaging and appealing to an increasing number of viewers, thereby heightening the reach of content to inform practice.

Many stressors and triggers were reviewed across the widely viewed videos, several of which included social determinants of health. In addition, all of the principles of trauma-informed care (safety, trustworthiness, empowerment, collaboration, and choice) were covered across these videos. Multiple stressors and triggers stemmed from formative experiences in childhood (e.g., adverse childhood experiences, grief/loss, and violence), as presented in this sample of videos. It follows that drawing on one or more resources and strategies that integrates one or more principles of trauma-informed care reviewed among the videos could inform the development of performance and treatment plans to work through barriers, stressors, and triggers in any context with employees, students, and patients to create a path for healing, productivity, a culture of safety, resilience, and so much more. These were some of the widely covered benefits of trauma-informed care presented in the widely viewed videos. It follows that the diversity of resources and strategies, along with the range of benefits presented in the videos, collectively yield promise in the versatility and adaptability of trauma-informed approaches to address unique and diverse circumstances on both individual and community levels. On a clinical level, this could also be further evaluated for consideration of integrating individual and group counseling informed by trauma-informed principles as part of the constellation of interventions covered by health insurance plans for sustainability in treatment. In the meantime, the depth and breadth of resources and strategies presented on social media could be a helpful starting point for healthcare organizations, academic institutions, and other professional workplace settings to bolster trust, safety, and resilience for patient, student, and employee populations.

Notably, there was scant coverage of diversity, equity, and inclusion across these widely viewed videos on trauma-informed care. Each of these principles is an integral part of bolstering a culture of safety, resilience, and collaboration along with building relationships guided by trust and transparency (Morgan et al., 2023). As the future of DEI initiatives remains unclear in the United States, it is ever more imperative to elucidate the significance of DEI in informing effective treatment planning and thriving organizations that provide spaces for healing through trauma and bolstering mutual respect and collaboration to yield a larger impact on both community and clinical levels. It is crucial to consider integrating future content that presents how DEI principles can also represent outcomes from the implementation of trauma-informed practices in mobilizing more robust workforces, care delivery models, and healthier communities.

Trauma-informed principles are also utilized to build professionalism (e.g., leadership, administrative skills, problem-solving capabilities, change agents) in the workforce context to affect positive change with care delivery models and across organizational culture (Koloroutis & Pole, 2021; Houlihan et al., 2024; Papa & Robinson, 2023; Harris et al., 2024). Notably, professional roles guided by trauma-informed approaches were not substantially covered across the widely viewed videos. It follows that future content on trainings and curriculum could integrate focus areas on building these critical roles to create more successful workforces that support professional growth and development in the context of cultivating a culture of safety, trust, collaboration, mutual respect, resilience, and DEI.

There were both strengths and limitations in this descriptive, observational study. First, both inter-rater and intra-rater reliability were substantially high, thereby increasing the precision of the coding instrument in collecting data on sources, formats, and content among the widely viewed videos. In addition, this study has a moderate sample size of 100, which was sufficient for identifying widely covered and uncovered content as the basis to assess for trends, patterns and representation of content in line with guidelines, clinical approaches, and training programs. One limitation of this study was related to the cross-sectional study design which involved extracting videos at one conceptual point in time, thereby limiting the replicability of this study. Specifically, the chronology of widely viewed videos on social media may change with the publication of new content, which, in turn, could further impact the level of engagement (e.g., changes in engagement metrics such as view counts, likes, tweets, shares, comments). In addition, this study solely utilized view count as the engagement metric, and future studies could draw on other prevalent ones to assess engagement among viewers. Another limitation of the present study was that only videos in English were included for review and analysis, thereby precluding content that could be pertinent and highly engaging in a different language. Nevertheless, the culmination of content from these videos demonstrated the far-reaching impact of trauma-informed care and, in turn, yielded significant potential for building on these practices across a multitude of systems worldwide.

## 5. Conclusions

Trauma-informed care is a rising, trending clinical and organizational approach that transcends a range of contexts, including healthcare systems, academic institutions, prisons, and workforces. Much of the most popular content on social media pertaining to trauma-informed care is in line with institutional guidelines worldwide. Nongovernmental sources lead in posting this content, and there are certainly opportunities to engage the government on federal, state and local levels in disseminating content on trauma-informed care that could be engaging, appealing and relatable to a wide range of audiences. There are ample organizational and healthcare implications from harnessing the potential of social media as a communication medium in presenting content on trauma-informed care that can be adapted across diverse settings. Social determinants of health and DEI considerations are at the heart of shaping the delivery of trauma-informed care on both individual and community levels. Furthermore, multiple trainings and curriculum that were covered in the widely viewed videos could likely be tested further across institutional programs to cultivate a trauma-informed workforce that yields productivity, trust, collaboration, safety, and mutual respect. In light of our political climate, a lot of work remains in regard to establishing trauma-informed practices as an integral part of effective treatment planning and care delivery, which are formative in thriving organizations and at the heart of creating safe and healthy communities worldwide.

## Figures and Tables

**Table 1:** Frequencies, view counts, and cumulative view count percent of widely viewed trauma-informed care videos by upload source.

Source	N	View Count	Cumulative View Count Percent (%)
Nongovernmental/Organizational	75	12,283,746	75.9
Other	13	3,651,644	22.56
Government	7	178,666	1.1
Consumer	5	69,556	0.43

**Table 2:** Frequencies, view counts, and cumulative view count percent of widely viewed trauma-informed care videos by format.

Format	N	View Count	Cumulative View Count Percent (%)
Other Formats	49	14,201,211	87.75
Animation	30	5,900,868	36.46
Talk by Professional	70	2,935,155	18.14
Interview	3	1,510,674	9.33
Demonstration/Experiment	7	1,382,570	8.54
Still Images	20	863,327	5.33
Testimonial	8	677,147	4.18
Multiple Formats	6	553,553	3.42
TV Talk Show/Discussion Panel	1	304,006	1.88
Advertisement	1	16,942	0.1
News Report with Anchor	1	2033	0.01
Documentary	0	0	0
V-Blog	0	0	0

**Table 3:** Frequencies, view counts, and cumulative view count percent of widely viewed trauma-informed care videos by stressors/triggers.

Stressors/Triggers	N	View Count	Cumulative View Count Percent (%)
Trauma	78	15,272,845	94.37
Grief/Loss	8	7,838,984	48.44
Psychiatric Co-Morbidities	31	5,943,040	36.72
Adverse Childhood Experiences (ACE)	33	5,712,561	35.3
Violence	22	5,551,070	34.3
Environmental Adversity	18	5,318,265	32.86
Abuse	25	5,248,570	32.43
Domestic Violence	15	5,003,393	30.92
Neglect	17	4,437,854	27.42
Substance Use Disorders	20	2,983,672	18.44
Increased Suicide Risk	8	2,116,616	13.08
Sexual Abuse	14	2,096,372	12.95
PTSD	6	1,835,617	11.34
Poverty	10	1,270,190	7.85
Chronic Diseases	7	953,397	5.89
Health Co-Morbidities	5	932,058	5.76
Discrimination	5	704,266	4.35
Physical Abuse	10	623,189	3.85
Chronic Stress	4	533,747	3.3
Increased Healthcare Utilization	4	524,911	3.24
Emotional Abuse	6	490,080	3.03
Occupational Adversity	1	198,356	1.23

**Table 4:** Frequencies, view counts, and cumulative view count percent of widely viewed trauma-informed care videos by resources and strategies.

Resources and Strategies	N	View Count	Cumulative View Count Percent (%)
Self-Regulation	19	4,503,796	27.83
Respond (Don’t React)	18	3,778,173	23.35
Training	46	2,570,491	15.88
Stress Management	20	1,262,564	7.8
Emotionally and Psychologically Safe Space	23	945,941	5.85
Trauma-Informed Workforce	13	877,520	5.42
Coaching and Mentoring	14	804,306	4.97
Community Resources	10	674,426	4.17
Screening	15	588,842	3.64
Active Listening	7	534,465	3.3
Communication	7	351,031	2.17
Counseling	8	324,864	2.01
Literature	7	218,536	1.35
Guidelines	9	150,726	0.93
Policies/Legislation	9	144,792	0.89
Self-Reflection	1	103,827	0.64
Role Playing	3	89,094	0.55
Support Group	1	84,856	0.52
Pamphlets, Leaflets, Booklets	4	38,936	0.24
Crisis Intervention	4	24,521	0.15
Workgroup	1	4966	0.03
Internal Organization Resources	1	3774	0.02
Campaign	0	0	0
Flexibility	0	0	0
Webinar	0	0	0
In-Service	0	0	0

**Table 5:** Frequencies, view counts, and cumulative view count percent of widely viewed trauma-informed care videos by benefits.

Benefits	N	View Count	Cumulative View Count Percent (%)
Mutual Respect	13	8,275,392	51.13
Healing	39	5,939,693	36.7
Increased Safety	51	5,433,443	33.57
Healthier Relationships	34	5,109,138	31.57
Enhanced Trust	30	5,061,718	31.28
Effective Treatment Planning	13	3,880,541	23.98
Resilience	20	3,834,548	23.69
Thriving Organization	20	853,184	5.27
Increased Empowerment/Self-Efficacy	17	729,658	4.51
Optimizing Learning Outcomes	16	689,752	4.26
Productivity	13	648,191	4
Improved Quality and Delivery of Care	20	319,322	1.97
Build Leadership Skills	2	265,652	1.64
Improved Health Outcomes	6	249,210	1.54
Improved Performance	10	112,679	0.7
Conflict Resolution	6	107,905	0.67
Teamwork	5	37,181	0.23
Improved Decision-making	0	0	0
Efficiency	0	0	0

## Data Availability

The original contributions presented in this study are included in the article. Further inquiries can be directed to the corresponding author.
